# Six Letters with Answers

**Published:** 1881-10

**Authors:** 


HISEVILLE, KY., Aug. 2, 1881.
DR. THAD S. UP DE GRAFF:

*My Dear Sir:*—Your ever welcome journal—THE BISTOURY, came to hand last week, as sprightly and full of interesting matter as ever. I see by it that you have been away on your annual fishing tour. I hope to come north next season, and I want you to instruct your pickets not to fire on a six foot, Italian looking, Kentucky Doctor, if perchance he should run upon them in seeking your camping grounds. Dr. I would be exceedingly well pleased to meet you in your quiet, shady retreat. * * T. L. W.

Saddle that Kentucky mare of yours, start in season, when you will find us as usual at the old camping grounds ready to welcome you any time during the month of June. We have instructed Fritz not to lasso any six footer that he may find prowling about the outskirts of the camp.

---

POTTSDAM, N. Y., Sept. 12, 1881.
DR. UP DE GRAFF:

Is the wine beef and iron advertised in your journal by Warner & Co., what it is represented to be? * * * J. D.

We often have letters like the above making inquiries about preparations advertised in this journal. We desire it to be understood that we do *not* advertise patent medicines or any other worthless medicines, although offered extravagant prices so to do. We have but six advertising pages and do not mean to increase the number. These we keep filled, without difficulty, with advertisements of goods of genuine merit. We accept no others.

---

CANANDAIGUA, N. Y., Aug. 20, 1881.
DR. UP DE GRAFF:

There is a "Doctor Myers" operating along the line of the Central road selling spectacles and represents himself as your agent. He is doing a thriving business on such representation. I think you have warned people that you have no agents. Am I right? J. F. M.

We have repeatedly warned the public against persons representing themselves as "agents" of ours in selling spectacles. We do not need nor employ agents of any sort. Any one who so represents himself is a fraud. Do not trust *any* traveling doctor or spectacle vender, no matter what his representations are. If such persons are trustworthy, they will have sufficient patronage at home, without perambulatlng the country "drumming up" customers. We are exceedingly obliged to the good people for a seeming confidence in us that would warrant them in making purchases of traveling humbugs who represent themselves as our "agents," but why they should think it necessary for us to employ "agents," is beyond our comprehension.

---

TUSCUMBIA, Ala., Sept. 1, 1881.
DEAR BISTOURY:

I read what you had to say of the assassin Giteau, in the last BISTOURY, Suppose they make him out insane, what should then be done with him? J. D.

Well, our impression is, that the fellow should go free. But, we should like to have it clearly specified at what particular hour he would leave the prison. Our impression is that he would thus better meet his deserts and spare the country considerable expense.

---

SYRACUSE, N. Y., Aug. 30, 1881.
DEAR BISTOURY:

I enclose my subscription for the ensuing two years, because it is more convenient to send a dollar bill than a half dollar in silver or postage stamps, beside, I think the BISTOURY is worth more than you ask for it, so that you deserve to be paid for a long while in advance. Will you please send me a prescription for freckles and much oblige. J. D. F.

Our correspondent will find two excellent prescriptions in our Medical Items and News.

---

HARVE DE GRACE, Md., July 30, 1881.
DR. UP DE GRAFF:

Dear Sir:—I feel as though I must write and thank you heartily for teaching us the way to sensibly and healthfully enjoy our vacation. You see, we, that is, the boys and I—read your book "Bodines." We made our tent and boat according to your instructions. Yes, we even went into the tin-ware scheme, and must say that everything worked admirably. We camped on the Susquehannah during the whole of July, wife, myself and four children. We fished for bass—as you do for trout—with a fly, and a right good vacation we had. Heretofore we have gone to so-called summering places, at a great expense, much fatigue and without special gain in rest or health. Camping out, captivated us, and we mean to keep it up every summer as long as we live. By the way, Mrs. B. used no other cook-book but "Bodines." She pronounces it "splendid"—and that means the ultimate degree of praise for a woman.

Yours, heartily, P. R. B.

We thank our friend for his good letter and hearty appreciation of camp life. We are sure that this method of spending a summer vacation need only be tried to be at once approved and thence-after always indulge in.

